# Investigation of the Impact of Manufacturing Methods on Protein-Based Long-Acting Injectable Formulations: A Comparative Assessment for Microfluidics vs. Conventional Methods

**DOI:** 10.3390/pharmaceutics16101264

**Published:** 2024-09-27

**Authors:** Nihan Yonet-Tanyeri, Robert S. Parker, Louis D. Falo, Steven R. Little

**Affiliations:** 1 Department of Chemical Engineering, University of Pittsburgh, 940 Benedum Hall, 3700 O’Hara Street, Pittsburgh, PA 15213, USA; niy14@pitt.edu (N.Y.-T.); rparker@pitt.edu (R.S.P.); 2 Department of Bioengineering, University of Pittsburgh, 302 Benedum Hall, 3700 O’Hara Street, Pittsburgh, PA 15213, USA; lof2@pitt.edu; 3 Department of Critical Care Medicine, University of Pittsburgh, 3550 Terrace Street, Alan Magee Scaife Hall, Suite 600, Pittsburgh, PA 15213, USA; 4 Department of Clinical and Translational Science, University of Pittsburgh, Forbes Tower, Suite 7057, Pittsburgh, PA 15213, USA; 5 McGowan Institute for Regenerative Medicine, University of Pittsburgh, 450 Technology Drive, Suite 300, Pittsburgh, PA 15219, USA; 6 Department of Dermatology, University of Pittsburgh School of Medicine, 3708 Fifth Avenue, Pittsburgh, PA 15213, USA; 7 Department of Pharmaceutical Sciences, University of Pittsburgh, 3501 Terrace Street, Pittsburgh, PA 15213, USA; 8 Department of Ophthalmology, University of Pittsburgh, 203 Lothrop Street, Pittsburgh, PA 15213, USA; 9 Department of Immunology, University of Pittsburgh, 200 Lothrop Street, Pittsburgh, PA 15213, USA

**Keywords:** biologics, long-acting injectables, microparticles, protein-based drug formulations, microfluidics, drug delivery, biodegradable polymers, controlled drug release

## Abstract

**Background/Objectives:** Microparticle-based drug delivery systems offer several advantages for protein-based drug formulations, enhancing patient compliance and therapeutic efficiency through the sustained delivery of the active pharmaceutical ingredient. Over the past few decades, the microfluidics method has emerged as a continuous manufacturing process for preparing drug-encapsulating microparticles, mainly for small molecule drugs. However, comparative assessments for the conventional batch method vs. the microfluidics method for protein-based drug formulations have been lacking. The main objective of this study was to generate immunomodulatory protein drug-loaded injectable formulations using both conventional batch and microfluidics methods. **Methods**: Therefore, rhCCL22-loaded poly(lactic-co-glycolic) acid (PLGA) microparticles were prepared by conventional homogenization and microfluidics methods. **Results**: The resulting microparticles were analyzed comparatively, focusing on critical quality attributes such as microparticle size, size distribution, morphology, drug encapsulation efficiency, release kinetics, and batch-to-batch variations in relation to the manufacturing method. Our results demonstrated that the conventional method resulted in microparticles with denser surface porosity and wider size distribution as opposed to microparticles prepared by the microfluidics method, which could contribute to a significant difference in the drug-release kinetics. Additionally, our findings indicated minimal variation within batches for the microparticles prepared by the microfluidics method. **Conclusions**: Overall, this study highlights the comparative assessment of several critical quality attributes and batch variations associated with the manufacturing methods of protein-loaded microparticles which is crucial for ensuring consistency in efficacy, regulatory compliance, and quality control in the drug formulation manufacturing process.

## 1. Introduction

Biologics, such as peptides and proteins, face several challenges in terms of effective delivery to the body. They typically have short half-lives in body fluids, leading to their rapid elimination. This may necessitate multiple drug administrations, posing a significant barrier to patient adherence [1]. Additionally, the oral administration route of protein drugs presents another major obstacle for clinical success. The relatively large size of the protein molecules causes permeability issues in the gastrointestinal track. Furthermore, their enzymatic degradation in the gastric environment, in particular at low pH, leads to limited stability and a loss of therapeutic activity [2]. In this regard, drug delivery systems, specifically peptide/protein-encapsulating long-acting injectables, offer enhanced drug retention, improved patient compliance, local delivery opportunity, and better clinical outcomes through their controlled release characteristics in the body [3,4].

Protein-encapsulating long-acting injectable formulations have been widely studied in the form of micro- and nanoparticles. Among these formulations, microparticle-based formulations offer distinct advantages, serving as a continuous depot for protein drugs and maintaining sustained delivery over extended periods in vivo. One of the main reasons for this is that microparticles, typically greater than 10 μm, enhance the bioavailability of the protein drug by reducing the risk of phagocytosis and rapid clearance from the body [5]. In this context, the upper size limit of microparticles for long-acting injectable formulations is determined by the route of administration, requiring compatibility with appropriate needle sizes, as well as ensuring the microparticle concentration that keeps the drug substance within the therapeutic window. Additionally, microparticles can be administered directly to the site of action, such as a cancer microenvironment, inflamed, infected, or wounded areas, which improves drug efficacy, reduces systemic side effects, and allows for lower overall doses [6]. Biodegradable polymers, such as poly(lactic-co-glycolic) acid (PLGA), can also be utilized to generate microparticles with long-acting features, providing tunable degradation characteristics and, accordingly, drug-release kinetics [7,8,9,10]. These types of formulations have conventionally been manufactured using batch manufacturing processes using emulsions or spray drying [11,12].

A continuous, microfluidics-based manufacturing method has emerged as a powerful tool in drug delivery systems and is increasingly employed to produce microparticle-based drug formulations [13,14]. Specifically, microfluidics technology offers precision and control over critical process parameters, such as flow rates and droplet configuration, to create uniform droplets that are then solidified to form microparticles [15,16]. Compared to conventional methods for microparticle production, such as emulsification, the microfluidics method enables a continuous, reproducible, and scalable production platform for drug delivery systems [17]. In addition, the microfluidics method provides advantages over other continuous manufacturing processes used for drug-loaded microparticle production. For example, hot melt extrusion offers continuous production capacity for protein-loaded drug product formulations but requires high temperatures and pressures, which can lead to a loss in protein stability [18,19]. In contrast, the microfluidics method allows for milder processing conditions than hot melt extrusion, potentially helping protein drugs maintain their therapeutic function [20]. Furthermore, the implementation of additive manufacturing technology in the pharmaceutical field has led to continuous drug product manufacturing via the 3D printing of microparticles, which is primarily suitable for small-scale applications [21]. Despite the expanding utilization of microfluidics technology in formulating drug delivery systems within the pharmaceutical field, examples of microfluidics-assisted production have been largely limited to hydrophilic small molecules or poorly water-soluble drugs [17,22,23]. Therefore, further investigations are needed to explore the capacity of the microfluidics method in comparative studies with the conventional emulsification method, especially for protein-based drug formulations [24,25,26,27,28].

In this study, we assess the impact of the manufacturing methods—the conventional batch vs. continuous microfluidics methods—on protein-based long-acting injectable formulations in a comparative study. Specifically, we generated protein-based drug formulations using a generally recognized as safe (GRAS) biodegradable polymer, poly(lactic-co-glycolic) acid (PLGA), to encapsulate a protein drug, recombinant human CCL22 (rhCCL22) [29]. rhCCL22 is known as an immunomodulatory chemokine found in the body. A gradient of this chemokine recruits regulatory T cells to a local site and can suppress inflammation when locally administered to inflamed tissue, such as periodontitis (gum disease) or organ transplantation animal models [30,31,32]. The rhCCL22-containing PLGA microparticles were prepared using both conventional emulsification and microfluidics methods. We investigated selected critical quality attributes of these drug formulations, including microparticle size, size distribution, microparticle surface morphology, inner porosity, rhCCL22 entrapment efficiency (%), and the release kinetics for each manufacturing method. Ultimately, we compared these manufacturing methods in terms of batch-to-batch variations, considering these critical quality attributes.

## 2. Materials and Methods

### 2.1. Materials

Poly(lactic-co-glycolic) acid (PLGA; Resomer RG502H, 50:50 lactic/glycolic acid, MW: 7000–17,000 kDa), the polymer used for the preparation of microparticles, was purchased from Sigma-Aldrich (St. Louis, MO, USA). Sodium chloride (NaCl), dichloromethane (DCM), phosphate-buffer saline (PBS), and sodium dodecyl sulfate (SDS) (10% solution) were obtained from Fisher Scientific (Waltham, MA, USA). Polyvinyl alcohol (PVA; MW ~25%, 98% hydrolyzed) was supplied by Polysciences (Warrington, PA, USA). Recombinant human CCL22 (rhCCL22; 69 a.a.) was purchased from PeproTech (Cranbury, NJ, USA). Bovine serum albumin (BSA) and enzyme-linked immunosorbent assays (ELISAs, rhCCL22) were obtained from R&D systems (Minneapolis, MN, USA).

### 2.2. Methods for Preparation of rhCCL22-Loaded PLGA Microparticles

#### 2.2.1. rhCCL22-Loaded PLGA Microparticles via Conventional Method

**Figure 1:**
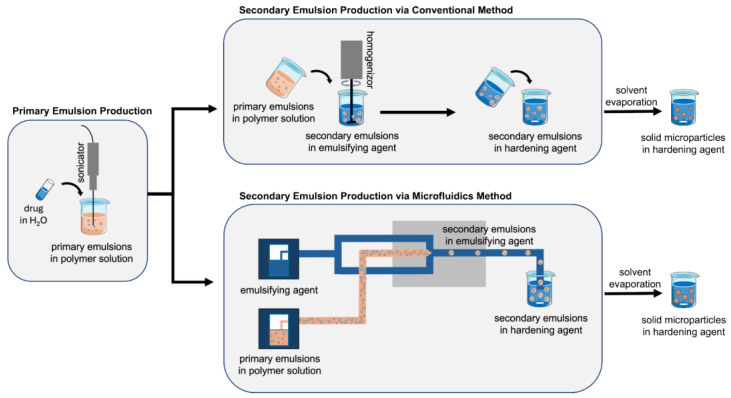
Schematic illustration of the manufacturing methods for protein-loaded PLGA microparticles using microfluidics and conventional methods.

rhCCL22-loaded PLGA microparticles were prepared by the conventional method similar to that previously described (Figure 1) [30,31]. Briefly, the primary emulsion of rhCCL22-containing water droplets was generated by sonicating (EpiShear probe sonicator, Active Motif, Carlsbad, CA, USA) (55% amplitude) 200 μL of DI water in 4 mL of 5% PLGA (200 mg) solution in DCM for 10 s. The water phase was composed of 25 μg of rhCCL22, 10 mg/mL of BSA and 15 mM of NaCl. This primary emulsion was poured into 60 mL of aqueous 2% PVA solution (emulsifying agent) and homogenized (Silverson L4RT-A) at 2500 rpm for 1 min to form the secondary emulsion. Then, the secondary emulsion was poured into 80 mL of aqueous 1% PVA solution (hardening agent). This mixture was stirred for 3 h at room temperature at 600 rpm (Telesystem HP15 RM, Variomag, Daytona Beach, FL, USA) to evaporate DCM. Solidified rhCCL22-loaded PLGA microparticles were then washed with DI water for four times to remove the residual emulsifying/hardening reagent, PVA. Finally, microparticles were frozen in liquid nitrogen, lyophilized (Benchtop Pro, VirTis SP Scientific, Warminster, PA, USA) for 48 h and kept at −20 °C for extended storage. Ultimately, this production procedure was repeated two more times to generate 3 different batches.

#### 2.2.2. rhCCL22-Loaded PLGA Microparticles via Microfluidics Method

rhCCL22-loaded PLGA microparticles were prepared by a microfluidics method using a microfluidic chip with a 3D flow-focusing cross-junction design (Dolomite 3200433) (Figure 1) [17]. Initially, the primary emulsion of rhCCL22 water droplets was prepared by the same method described in Section 2.2.1. Then, this primary emulsion and the aqueous solution of 2% PVA (emulsifying agent) were mobilized into the microfluidic chip using corresponding pressure pumps (Dolomite Mitos 3200175, Dolomite Microfluidics, Royston, UK). The flow rates for each fluid, the primary emulsion and the emulsifying agent, were monitored by flow sensors, (1–50 µL/min) (Dolomite Mitos 3200098) and (30–1000 µL/min) (Dolomite Mitos 3200097), respectively. The secondary emulsions in terms of rhCCL22-containing PLGA droplets were formed at the cross-junction under certain flow conditions to maintain the dripping regime [33]. Specifically, the flow rate of the primary emulsion phase was set to 7 μL/min while the flow rate of the emulsifying agent was set to 85 μL/min. These droplets were continuously collected in the aqueous solution of 1% PVA (hardening agent) for 3 h while the mixture was under stirring conditions at 600 rpm. Then, this mixture was stirred for 3 h at room temperature at 600 rpm to evaporate DCM. Solidified rhCCL22-loaded PLGA microparticles were then washed with DI water four times to remove the residual emulsifying/hardening reagent, PVA. Finally, microparticles were frozen in liquid nitrogen, lyophilized for 48 h and kept at −20 °C for extended storage. Ultimately, this production procedure was repeated two more times to generate 3 different batches.

### 2.3. Methods for Characterization of Primary Emulsion Stability

It is critical to assess the stability of the primary emulsion, especially considering the extended period (3 h) of the secondary emulsion (PLGA droplets) production in the microfluidics method. Therefore, the primary emulsion, rhCCL22-containing water droplets in the PLGA solution, was generated as described in Section 2.2.1. In order to compare the droplet size change over time in the absence of rhCCL22, the same volume of DI water (200 µL) containing 10 mg/mL of BSA and 15 mM of NaCl was used to prepare the rhCCL22-free primary emulsion. Then, the droplet size of the primary emulsion (with or without rhCCL22) was measured using dynamic light scattering (Zetasizer nano317, Malvern, UK) immediately following the sonication process and after waiting for 3 h at room temperature to represent the microfluidic-assisted droplet collection time (see Section 2.2.2). Each measurement was taken in triplicate, and the result was shown the average droplet size for 3 replicating samples.

### 2.4. Characterization of rhCCL22-Loaded PLGA Microparticles

rhCCL22-loaded PLGA microparticles were prepared by two different manufacturing methods—conventional and microfluidics methods. Initially, the primary emulsion of protein-containing water droplets was formed in the PLGA solution. Then, this primary emulsion was used to generate the secondary emulsion using either homogenization or microfluidics. The resulting microparticles were characterized the same way regardless of the manufacturing method, whether conventional or microfluidics.

#### 2.4.1. Particle Size Measurement

To determine the effects of the manufacturing method (conventional vs. microfluidics) on the rhCCL22-loaded PLGA microparticle size, volume impedance measurements (Multisizer-3, Beckman Coulter Counter, Brea, CA, USA) were conducted using lyophilized microparticles. For this purpose, each measurement was carried out for at least 10,000 counts of microparticles. The differential volume-weighted (%) size distribution was reported. To explore the batch-to-batch variation in the microparticle size distribution regarding the manufacturing method type, each batch was analyzed.

#### 2.4.2. Imaging Particle Morphology and Inner Porosity

The surface morphology and the inner porosity of rhCCL22-loaded PLGA microparticles that were prepared via the conventional or microfluidics methods were investigated using the scanning electron microscopy (SEM) imaging technique (Zeiss, Jena, Germany). Briefly, lyophilized PLGA microparticles were mounted onto a sample holder using double-sided conducting (carbon or copper) tape. Then, a 5–10 nm-thick gold palladium layer was sputter-coated (Denton Sputter Coater, Moorestown, NJ, USA) on the sample. Electron micrographs for the area of interest were obtained at 250× magnification to capture the surface morphology of the microparticles. Sample preparation for imaging the inner porosity of the microparticles is slightly different. Before coating the sample with the conducting layer, microparticles were randomly fractured using a razor blade. Electron micrographs of the fractured cross-sections magnification were obtained at 2.15 kx.

#### 2.4.3. rhCCL22 Encapsulation Efficiency (%)

The change in the amount of rhCCL22 encapsulated in PLGA microparticles as a function of the manufacturing method, conventional vs. microfluidics, was evaluated by extracting rhCCL22 from the microparticles and further quantified using ELISA. Briefly, ~3–5 mg of rhCCL22-loaded PLGA microparticles (*n* = 3) was vortexed in 500 μL of DCM to completely dissolve PLGA. A total of 250 mL of 0.1% SDS containing PBS was then mixed with this organic phase and vortexed. The two-phase mixture was centrifuged at 5000 rcf for 10 min to phase separate the liquids. Then, the top aqueous portion was removed. This extraction process was repeated two more times to remove the remaining rhCCL22 from the DCM phase. Finally, the collected aqueous phase was used in ELISA to determine the rhCCL22 concentration which was normalized to the total weight of PLGA microparticles used in the extraction. The entrapment efficiency (rhCCL22 entrapment (%)) was then calculated by dividing the rhCCL22 amount that was detected in one mg of PLGA microparticles (We) by the total amount of rhCCL22 per mg of PLGA (initial polymer weight) that was originally used during microparticle preparation (Wt), multiplied by 100.

% EE = (We/Wt) × 100(1)

#### 2.4.4. In Vitro rhCCL22 Release Study

The in vitro release study was performed by suspending (~5 mg) rhCCL22-loaded PLGA microparticles in 1 mL of 1% BSA containing PBS (drug-release medium) and incubating suspensions on an end-to-end rotator (Thermo Fisher) at 37 °C. These suspensions were then centrifuged at certain time intervals for 21 days of the release study to remove the supernatant. In each time interval, the fresh drug-release medium with the equivalent volume of the removed supernatant was added onto microparticles to resuspend while maintaining the total volume of 1 mL. The rhCCL22 concentration in the collected supernatants was quantified by ELISA. Then, the cumulative rhCCL22 release (%) was calculated by considering the encapsulated rhCCL22 amount per mg of PLGA microparticles as 100%. To investigate possible variations in the rhCCL22 release kinetics within different batches, the release study was performed using three different batches.

#### 2.4.5. Batch-to-Batch Variation in the rhCCL22 Release Kinetics

To quantify the batch-to-batch variations in the in vitro rhCCL22 release kinetics, the release profiles were plotted using three batches of protein-loaded PLGA microparticles that were prepared via conventional and microfluidics methods. These profiles were fitted to the zero-order release model using the GraphPad Prism Software v10 (San Diego, CA, USA). As a result of this analysis, the release kinetics constant, k_0_, was determined for each batch. The lower and upper level 95% confidence intervals for each release constant were identified using the same software and further utilized for assessing the similarity/difference between batches for each manufacturing method.

#### 2.4.6. Statistical Analysis

GraphPad Prism Software v10 was used to plot the data for the primary emulsion droplet size, the differential volume-weighted (%) size distribution, rhCCL22 encapsulation (%), and the cumulative rhCCL22 release kinetics. Each measurement was repeated for three times and reported as the mean ± standard deviation (SD). To study the batch-to-batch variation in microparticle size distribution and the rhCCL22 release kinetics, three batches of rhCCL22-loaded PLGA microparticles were prepared for comparison using the conventional and microfluidics methods. GraphPad Prism Software v10 was also used to perform statistical analysis. A one-way ANOVA with Tukey post hoc test was performed to compare the mean of each experimental group. The statistical difference was determined based on the cutoff values of * *p* ≤ 0.05, ** *p* ≤ 0.01, *** *p* ≤ 0.001, **** *p* ≤ 0.0001.

## 3. Results and Discussion

### 3.1. Preparation of rhCCL22-Loaded PLGA Microparticles

It has been suggested in previous reports that microparticles can demonstrate size-dependent overall drug-release kinetics. For example, small microparticles can have a faster drug release than larger microparticles due to the larger surface area-to-volume ratio of the smaller particles [26,34]. Therefore, to assess the effects of the manufacturing methods—conventional vs. microfluidics—both as a comparative study and regarding the batch-to-batch variations when protein drug-release kinetics, controlling for average size, would aid in making comparisons on other critical quality attributes. Consequently, this study aimed to prepare protein-loaded microparticles within the same size range, with the manufacturing process parameters for both methods adjusted to minimize size disparities. In addition, long-acting formulations are typically designed with particle sizes that prevent phagocytosis, allowing for their retention at a local site (>1–10 μm) [5]. For this reason, rhCCL22-loaded PLGA microparticles larger than 10 μm were prepared, specifically targeting an average microparticle size between 20 and 30 μm, using both conventional and microfluidics methods.

Both manufacturing methods relied on a four-step production process (Figure 1). In the first step, the primary emulsion of rhCCL22-containing water droplets was formed in the PLGA solution. Then, the secondary emulsion of PLGA droplets was created in the emulsifying agent. In the third step, the PLGA droplets went through a solvent evaporation process in the hardening agent to solidify the droplets and subsequently form PLGA microparticles. Finally, the PLGA microparticles were washed with water to remove the emulsifying/hardening agent and then lyophilized to prepare the dry/powder form of the drug formulations. Among these four steps, all critical process parameters for every step were followed the same way for both manufacturing methods except for the second step, where the secondary emulsion was formed. For example, the sonication amplitude (55%) and duration (10 s) for the creation of the primary emulsion, the stirring speed (600 rpm) and duration (3 h) to harden the PLGA droplets, the centrifugation speed (10 G) and duration (10 min in each wash cycle of 4 runs), and the lyophilization period (48 h) were kept the same for both the conventional and microfluidics methods.

During these four steps, all solutions were used at the same concentrations when both manufacturing methods were utilized. For example, the rhCCL22 concentration (25 μg in 200 μL water), concentrations of the PLGA solution (50 mg/mL, 4 mL), the emulsifying agent (2% in water), and the hardening agent (1%, 80 mL) were kept the same regardless of the manufacturing method. Additionally, previous reports have shown the use of various excipients in the primary water droplets for various purposes [35]. For example, the inclusion of osmotic agent in the primary emulsion has been shown to improve the surface porosity of PLGA microparticles and subsequently improve protein-release kinetics [30]. Moreover, the addition of excipients in the water phase of the primary emulsion can increase protein stability. In this study, NaCl (15 mM) and BSA (10 mg/mL) were added into the water phase in the primary emulsion to serve as an osmotic agent and protein stabilizer, respectively. Overall, this study aims to prepare rhCCL22-loaded PLGA microparticles within a size range of 20 to 30 μm using both the conventional and microfluidics methods for comparative purposes, rather than identifying the more effective formulation. Therefore, formulation-related explorations and subsequent optimizations such as varying the concentrations of rhCCL22, PLGA, PVA, NaCl, and BSA were not included in this study.

One unavoidable difference between the conventional and microfluidics methods is the way the secondary emulsion is prepared. In the conventional method, the rhCCL22-containing primary emulsion was poured into the emulsifying agent and homogenized to prepare PLGA droplets. The homogenization speed and time were selected based on our previously published work and adjusted to 2500 rpm for 1 min, resulting in PLGA microparticles with an average size within the target range of 20–30 µm [30,31]. Conversely, the microfluidics method was employed so that the secondary PLGA droplets were generated at the cross-junction by flowing the primary emulsion and the emulsifying agent into the microfluidic chip. In this case, we referred to one of our previously reported works which mapped the relationship between the PLGA droplet size and the microparticle size under various flow rate conditions using the microfluidics method [33]. Based on these findings, the flow rates for the primary emulsion and the emulsifying agent were selected as 7 µL/min and 85 µL/min, respectively. Under these conditions, the PLGA droplets were generated at the proper size, and ultimately the resulting microparticles were prepared within the target size range of 20–30 µm.

### 3.2. Characterization of Primary Emulsion Stability

The microfluidic chip configuration had a single cross-junction for the secondary emulsion production for the microfluidics method, which resulted in a lengthy PLGA droplet collection process. This process collectively occurred over a period of hours (3 h) in the continuous process, compared to a very short homogenization step (1 min) for the conventional batch method. In this case, the primary emulsion stability during the PLGA droplet collection period would be the main concern for a successful drug encapsulation method. Therefore, we performed the droplet size measurements to assess any change in the primary emulsion water droplet size following the sonication process, particularly within a 4 h period, which is longer than the droplet collection period. We found that the average rhCCL22-containing water droplet size after sonication (395 ± 55 nm) did not significantly change (496 ± 301 nm) after 4 h. Similar measurements were carried out using rhCCL22-free water droplets as a negative control. Also, the change in the rhCCL22-free water droplet size (from 501 ± 75 nm to 589 ± 344 nm) within 4 h was not significant (Figure S1). These findings align with the literature reporting primary emulsion stability in the PLGA solution. Specifically, the primary emulsion of the water droplet stability was shown to increase as the PLGA concentration increased from 5 mg/mL to 35 mg/mL for a shorter period (100 min). The enhanced droplet stability in the concentrated PLGA solution was explained by an increase in the organic phase viscosity and a reduction in the interfacial tension [28]. In this regard, rhCCL22-containing primary emulsion stability in the PLGA solution for an extended period was an expected outcome, especially considering the much higher PLGA concentration we used in the primary emulsion (50 mg/mL).

### 3.3. Comparison of rhCCL22-Loaded PLGA Microparticle Size Measurements

**Figure 2:**
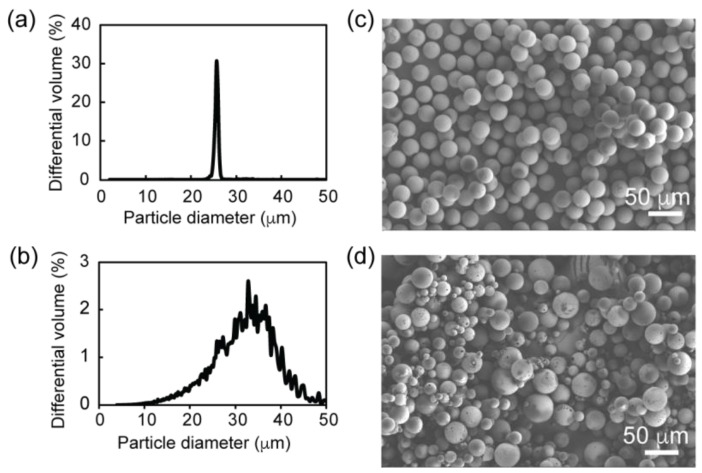
rhCCL22-loaded PLGA microparticles exhibit different size distribution based on the manufacturing method. (**a**,**b**) Volume-weighted size distributions and (**c**,**d**) representative scanning electron microscopy images of rhCCL22-loaded PLGA microparticles that were prepared by (**a**,**c**) microfluidics or (**b**,**d**) conventional methods.

rhCCL22-containing PLGA microparticles with a target average size within the 20–30 µm range were characterized in terms of average size and size distribution. The size measurements revealed that the PLGA microparticles generated via the conventional method had a broad size distribution (coefficient of variation: 25.0%) with an average size of 30.4 ± 7.6 µm. In contrast, the PLGA microparticles prepared by the microfluidics method showed a narrow size distribution (coefficient of variation: 6.5%) with an average size of 25.8 ± 1.7 µm. Scanning electron microscopy (SEM) images also highlighted the overall difference in the size distribution of the PLGA microparticles within a representative particle population (Figure 2).

### 3.4. Comparison of rhCCL22-Loaded PLGA Microparticle Surface Morphology

**Figure 3:**
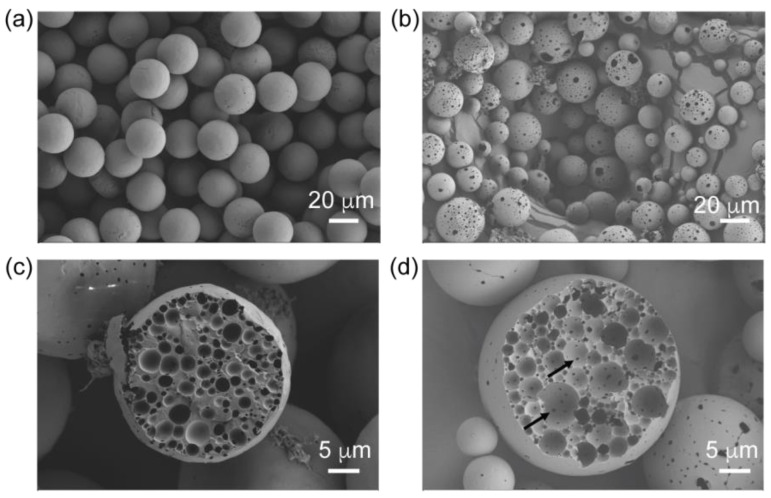
Representative scanning electron microscopy images demonstrate the surface morphology and inner porosity differences for rhCCL22-loaded PLGA microparticles. PLGA microparticles were prepared by (**a**,**c**) microfluidics and (**b**,**d**) conventional methods. Arrows in (**d**) highlight some examples of the inner pore connections.

Further characterization using SEM demonstrated subtle differences in the surface morphology and inner porosity of the PLGA microparticles that were prepared by the conventional and microfluidics methods (Figure 3). Even though the PLGA microparticles had a similar spherical 3D structure, the way that the microparticles were formed determined their surface characteristics. Specifically, the conventional method resulted in PLGA microparticles with more and larger surface porosity, while the microfluidics method produced PLGA microparticles with relatively less porosity with smaller surface pores (Figure 3a,b). This is an interesting result, especially considering the same kind and quantity of the osmotic agent was used in both manufacturing methods. It is likely that phase separation in the primary emulsion was maintained well during the secondary emulsion formation and the subsequent solvent evaporation process. Additionally, we observed more connected inner pore structures in the PLGA microparticles formed by the conventional method. Examples of the inner pore connections are highlighted with arrows in Figure 3d. However, the inner pores seemed less connected in the PLGA microparticles produced by the microfluidic manufacturing method due to the similar phase separation effect (Figure 3c,d).

### 3.5. Comparison of rhCCL22 Entrapment Efficiency (%)

In addition to emulsion stability, it is possible that the drug encapsulation efficiency may be affected by the extended period, whereby droplets continuously form in the microfluidics method. To assess the potential loss in the entrapment efficiency during this time, the 3 h-long secondary emulsion production process was divided into two parts. The first part included the collection of PLGA droplets for the first 1.5 h. These droplets were then stirred for an additional 1.5 h to complete the 3 h period. The PLGA microparticles generated from this first part are referred to herein as the early population. The PLGA droplet collection for the remaining 1.5 h are referred to herein as the late population. The PLGA droplets that were collected for the entire 3 h process were referred to as the mixed population. We quantified the rhCCL22 content in these early, late, and mixed populations to explore the effects of this long process on the rhCCL22 entrapment efficiency (%). The results showed no statistical difference between these parts regarding the protein entrapment efficiency (%). Thus, we confirmed that the microfluidics method produces the protein-loaded PLGA microparticles without any variation in the protein entrapment efficiency within the same batch (Figure S2).

**Figure 4:**
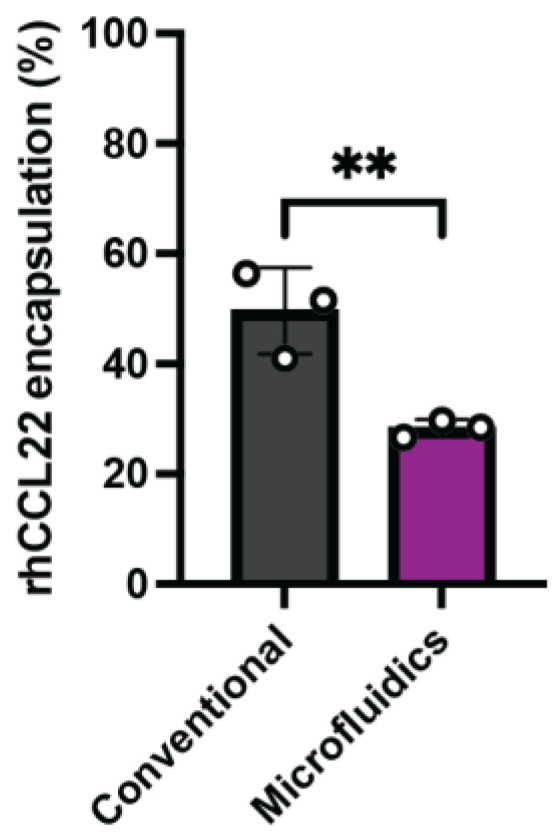
rhCCL22 encapsulation (%) in the PLGA microparticles shows a significant difference depending on the manufacturing process: microfluidics vs. conventional methods. ** *p* ≤ 0.01.

To assess the effects of the manufacturing method on protein entrapment efficiency, the same amount of rhCCL22 was included in the PLGA microparticles that were prepared using conventional and microfluidic methods. We observed a significantly different rhCCL22 entrapment efficiency (%) between the two different manufacturing methods (Figure 4). The average entrapment efficiency (%) was determined as 49.6 ± 7.9% (coefficient of variation: 15.8%) for the conventional method, while a lower average entrapment efficiency (%) of 28.3 ± 1.6% (coefficient of variation: 5.6%) was found for the microfluidics method. We hypothesize that this lower average entrapment efficiency may be related to a possible loss in the primary emulsions during the lengthy process, which takes 3 h to produce and collect secondary emulsions, as opposed to a much quicker homogenization process (1 min) for the conventional method. It is possible that the primary emulsions would aggregate during this time, as they remain in a non-mixing condition, and this subsequently would result in phase separation. This limitation could potentially be optimized in future work by the addition of a surfactant or co-emulsifier in the primary emulsion. Moreover, certain adjustments in the microfluidics system, such as modifying the microfluidic chip design to reduce the processing time for the production of secondary emulsions or altering the composition of the emulsifying agent, could possibly improve the entrapment efficiency.

### 3.6. Comparison of In Vitro rhCCL22 Release Kinetics

**Figure 5:**
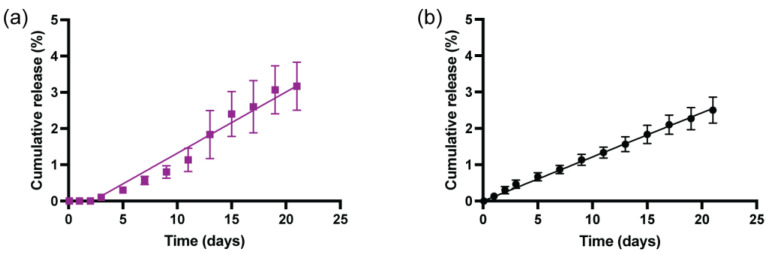
rhCCL22 release kinetics from PLGA microparticles demonstrates different release kinetics according to the manufacturing method. (**a**) Microparticles prepared by the microfluidics method showed a zero-order delayed (~2 days) release profile. (**b**) Microparticles prepared by the conventional method presented a zero-order release profile.

To study the effects of the manufacturing methods—conventional vs. microfluidic method—on the rhCCL22 release kinetics, an in vitro release assay was employed, using a rhCCL22-specific ELISA kit. The cumulative protein-release profiles within the first 21 days were utilized for this assessment (Figure 5). The duration of this protein-release study was selected based on earlier reports that showed rhCCL22-induced homeostasis specifically in the inflamed gum tissue (periodontitis) and the transplant microenvironment in corresponding animal models [31,32]. Our drug-release results demonstrated that both types of PLGA microparticles exhibited similar release behavior in terms of cumulative release (%) which is aligned with a previously published formulation that released rhCCL22 with a porous surface morphology [30]. Notably, a slightly different release pattern was observed during the first 9 days of the release study. The PLGA microparticles created by the microfluidics method showed a slightly slower release behavior compared to the microparticles generated by the conventional method. Specifically, no release was observed for the first 2 days, followed by a slower release kinetics until day 9 for microparticles prepared by the microfluidics method. This indicates zero-order with delayed release characteristics. However, the rhCCL22 release kinetics for PLGA microparticles generated by the conventional method followed what could be considered as near-zero-order release kinetics [36]. Moreover, representative fittings to the zero-order release model in Figure 5 (solid lines in Figure 5a,b) highlight a good fit for the zero-order release kinetics of the release profile for the conventional method, but a poor fit for the release profile of the microfluidics method. This result could be correlated with the previously mentioned surface porosity differences on the PLGA microparticles related to the manufacturing method (Figure 3). Denser and larger surface porosity on the PLGA microparticles could result in a zero-order trajectory for the conventional method, whereas fewer and relatively smaller surface pores on the PLGA microparticles could lead to a delayed (~2 days) and slower rhCCL22 release, followed by an increased release rate. Overall, we aimed to identify the potential differences in these protein-release kinetics in a comparative assessment that results from the manufacturing methods rather than optimizing the model predicting release kinetics. However, further studies could shed light on drug-release mechanisms, intraparticle structure change during degradation/dissolution, and the selection of the best model on the grounds of quality of fit and mechanistic explanation.

### 3.7. Comparison of Batch-to-Batch Variations between the Conventional and Microfluidics Methods

#### 3.7.1. rhCCL22-Loaded PLGA Microparticle Size and Size Distribution

**Figure 6:**
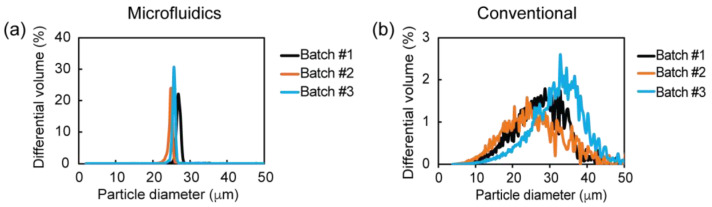
Batch-to-batch variations in the size distribution of rhCCL22-loaded PLGA microparticles when PLGA microparticles were prepared by (**a**) microfluidics and (**b**) conventional methods.

To identify potential variations in the microparticle size and size distribution within different batches, rhCCL22-loaded PLGA microparticles were prepared in three batches using both the conventional and microfluidics methods. The size measurements of each batch revealed that the average size of the PLGA microparticles prepared by both methods was within the target range of 20–30 µm (Figure 6). Specifically, three batches of the PLGA microparticles created by the microfluidics method had an average size of 25.8 ± 1.7 µm, 24.7 ± 1.7 µm, and 27.0 ± 1.8 µm. Similarly, the PLGA microparticles generated by the conventional method had an average size of 30.4 ± 7.6 µm, 23.4 ± 8.7 µm, and 25.3 ± 7.2 µm. However, the comparison of the batch variations between the manufacturing methods demonstrated a significant difference in terms of the size distribution of these batches. Notably, the microfluidics method resulted in a smaller variation in size within three batches with smaller coefficients of variation: 6.6%, 6.9%, and 7.7%, respectively (Figure 6a). Conversely, the conventional method yielded a broader size distribution within three batches, with larger coefficients of variation: 25.0%, 37.2%, and 28.5%, respectively (Figure 6b). A similar trend was observed in the overall size distribution of three batches for the microfluidics method, with a smaller coefficient of variation (6.6%) for the average size of three batches (25.8 ± 1.1 µm). Additionally, the average size of the three batches prepared by the conventional method showed a larger coefficient of variation (13.6%) for the average size of the three batches (26.4 ± 3.6 µm).

#### 3.7.2. rhCCL22 Entrapment Efficiency (%)

**Figure 7:**
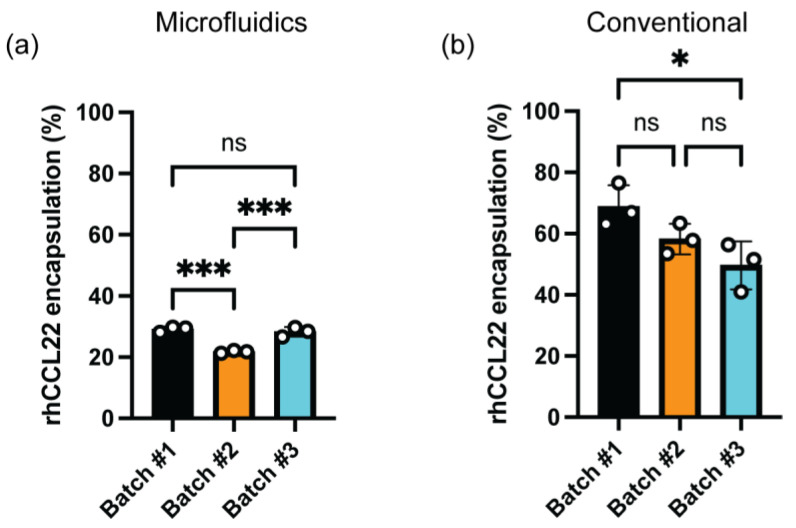
Batch-to-batch variations in terms of rhCCL22 encapsulation (%) in PLGA microparticles regarding the manufacturing method. rhCCL22-loaded PLGA microparticles were prepared by (**a**) microfluidics and (**b**) conventional methods. * *p* ≤ 0.05, *** *p* ≤ 0.001, ns indicates non-significant difference.

To assess any variation in the protein encapsulation efficiency (%) within different batches of the conventional and microfluidics methods, the PLGA microparticles discussed in the previous section were analyzed for encapsulation efficiency using ELISA. The rhCCL22 encapsulation efficiency (%) was found within the 20–40% range for three batches of the PLGA microparticles prepared by the microfluidics method. This value was higher, ranging in the 40–80% region for different PLGA batches formed by the conventional (batch) method. Overall, we observed limited differences within three batches for both manufacturing methods (Figure 7).

#### 3.7.3. In Vitro rhCCL22 Release Kinetics

**Figure 8:**
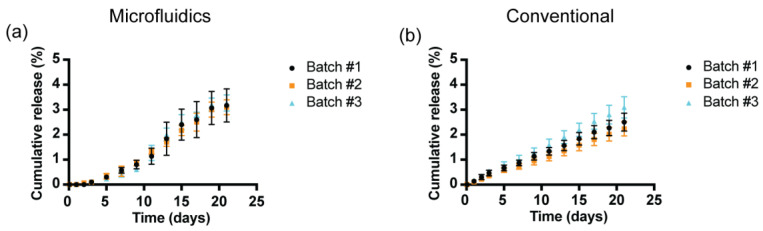
Batch-to-batch variations in terms of rhCCL22 release kinetics from PLGA microparticles. rhCCL22-loaded PLGA microparticles were prepared by (**a**) microfluidics and (**b**) conventional methods.

A comparison of the in vitro rhCCL22 release kinetics within these batches was investigated to explore the impact of the manufacturing method on the release behavior. Figure 8 demonstrates the cumulative rhCCL22 release (%) for different batches of the PLGA microparticles created by microfluidics and conventional methods. The cumulative release (%) of each batch prepared by both methods was less that 5% for the first 21 days of the release study, confirming the slow-release characteristics. Additionally, all three batches of the PLGA microparticles prepared by the microfluidics method showed zero release for the first 2 days and slower release kinetics until day 9, compared to the batches prepared using the conventional method. Moreover, the release kinetics for all batches prepared by the conventional method followed near-zero-order release kinetics.

Additional analyses were performed to quantify the similarity of the rhCCL22 release profiles between each batch for both manufacturing methods (Figures S3 and S4). Briefly, a linear regression was applied to each protein-release profile using the zero-order release model [36]. The best-fit value for each slope represented the rhCCL22 release constant, k_0_, for each batch. The quality of fit for a batch was computed using R^2^ between the batch release data and the zero-order model. Further, 95% confidence intervals of the estimated k_0_ parameter were compared to establish whether significant differences in the release rate existed between batches.

The computational analysis revealed that the three batches of the rhCCL22-containing PLGA microparticles generated by the conventional method had k_0_ values of 0.118, 0.1053, and 0.1453 (%) rhCCL22 per day (Figure S3). The model showed good fits with R^2^ values ranging between 0.9607 and 0.9633. Moreover, the k_0_ value for a specific batch (e.g., batch #1) was tested to determine if this value fell within the upper and lower levels of the 95% confidence intervals for the other batches (batch #2 and batch #3). We found that each k_0_ value was beyond the confidence intervals of the other batches. Therefore, we concluded that the differences between the k_0_ values within the three batches are significant.

Similar computational analyses were performed to determine the similarity of the rhCCL22 release profiles between the three batches of the rhCCL22-loaded PLGA microparticles created by the microfluidics method. Due to a delay in the rhCCL22 release during the first 2 days of the release study, estimations on the k_0_ value for each batch were performed by using linear regressions for a zero-order with delayed release kinetics. The calculated k_0_ values were 0.1671, 0.1625, and 0.174 rhCCL22 per day (Figure S4). Also, the R^2^ values were within the range of 0.9705 and 0.9922, implying good fits. Furthermore, each specific k_0_ value was within the confidence intervals of the other batches, indicating that the batch-to-batch variation in the rhCCL22 release kinetics is minimal.

In this study, we demonstrated that the microfluidics method produced rhCCL22-loaded PLGA formulations with minimal batch variations in drug-release kinetics, microparticle size distribution and drug encapsulation efficiency compared to the conventional method. However, several potential limitations must be addressed to scale up these formulations to industry applications. Specifically, the volume capacity of the primary emulsion and the emulsifying agent fed into the microfluidic chip is limited by the reservoir’s size. Furthermore, the continuous feeding of these liquids into a single-channel chip relies on the pump’s strength, which must mobilize liquids from large-volume reservoirs. Another limitation is the throughput of the droplet production process. Although droplet production speed could be improved by incorporating multiple microfluidic chips with several channels, innovative solutions will be necessary to address future throughput challenges.

## 4. Conclusions

Microfluidics technology has been increasingly utilized in the pharmaceutical field, especially for producing microparticle-based long-acting injectable formulations. However, there are only a few examples of drug-encapsulating microparticles generated by the microfluidics method for hydrophilic drugs. Therefore, further investigations into microfluidics-assisted protein-based long-acting injectable production would broaden the knowledge in the pharmaceutical field. This study explored the impact of the manufacturing methods—conventional batch vs. continuous microfluidic method—on protein-drug encapsulating long-acting injectable formulations. Our results revealed significant differences between these manufacturing methods. Specifically, the conventional method resulted in protein-encapsulating microparticles with larger and denser surface porosity, a broad particle size distribution, and higher encapsulation efficiency. Furthermore, the protein drug-release kinetics followed zero-order release kinetics. More importantly, the protein drug-release kinetics demonstrated variations between different batches, which could pose a predictability issue in therapeutic outcomes. However, microparticles generated by the microfluidics method resulted in uniform microparticles with low variation in size distribution, relatively less surface pore density and smaller surface pores. The protein encapsulation efficiency was found to be lower than that of microparticles prepared by the conventional method. Additionally, the protein drug-release kinetics showed a zero-order with delayed release kinetics. Overall, the microfluidics method resulted in the protein-based drug formulations with minimal variations between different batches, which could lead to more consistent performance in clinical practice.

## Data Availability

The raw data supporting the conclusions of this article will be made available by the authors upon request.

## Supplementary Materials

The following supporting information can be downloaded at: www.mdpi.com/article/10.3390/pharmaceutics16101264/s1, Figure S1: The change in the primary emulsion droplet size over 4 h period in the presence and absence of rhCCL22 in the water phase.; Figure S2: The long duration of microfluidics-assisted droplet production does not have an impact on the rhCCL22 encapsulation (%) for an early, late and mixed population of the collected droplets.; Figure S3: The quantification of the batch-to-batch variation in the conventional method for rhCCL22 release kinetics in terms of the zero-order release kinetics constant, k_0_.; Figure S4: The quantification of the batch-to-batch variation in the microfluidics method for rhCCL22 release kinetics in terms of the zero-order with delayed release kinetics constant, k_0_.
