# Supplementary material for: Investigation of the Impact of Manufacturing Methods on Protein-Based Long-Acting Injectable Formulations: A Comparative Assessment for Microfluidics vs. Conventional Methods
Source: Pharmaceutics. 2024 Sep 27;16(10):1264. doi: 10.3390/pharmaceutics16101264 (PMC11510299; doi:10.3390/pharmaceutics16101264)
Supplement: Supplementary file 1 [file pharmaceutics-16-01264-s001.zip › pharmaceutics-3230018-supplementary.pdf]

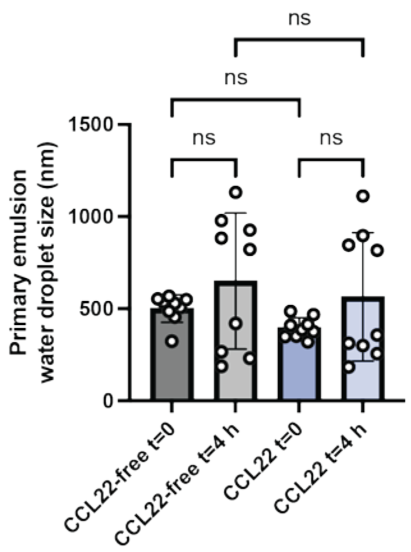

Figure S1. Change in the primary emulsion droplet size over 4 h period in the presence and absence of rhCCL22 in the water phase. The primary emulsion water droplet size measurements demonstrate that rhCCL22 containing water droplets remain intact in the PLGA solution for at least 4 hours during the microfluidics-assisted droplet production process. "ns" indicates not significant difference.

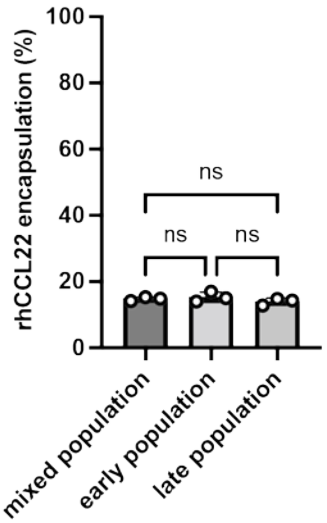

Figure S2. Long duration of microfluidic-assisted droplet production does not have an impact on the rhCCL22 encapsulation (%) for early, late and mixed population of the collected droplets.

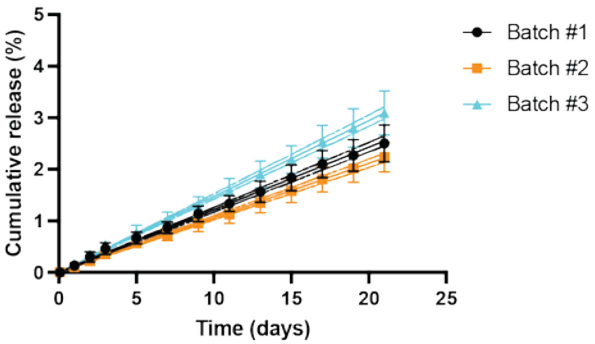

Equation for Zero-order drug release model: $M_t = k_0 t + b$

	Batch #1	Batch #2	Batch #3
k_0	0.118	0.1053	0.1453
Y-intercept	0.04959	0.007511	0.02791
X-intercept	-0.4203	-0.0713	-0.1921
R squared	0.9607	0.9619	0.9633
Lower Level 95% Confidence Interval	0.11	0.09836	0.1358
Upper Level 95% Confidence Interval	0.1259	0.1123	0.1547

Figure S3. Quantification of the batch-to-batch variation in the conventional method for rhCCL22 release kinetics in terms of the zero-order release kinetics constant, k_0. rhCCL22-loaded PLGA microparticles were prepared by the conventional method. The region between dashed lines color coded with the corresponding legend depicts the 95% confidence interval region for each batch.

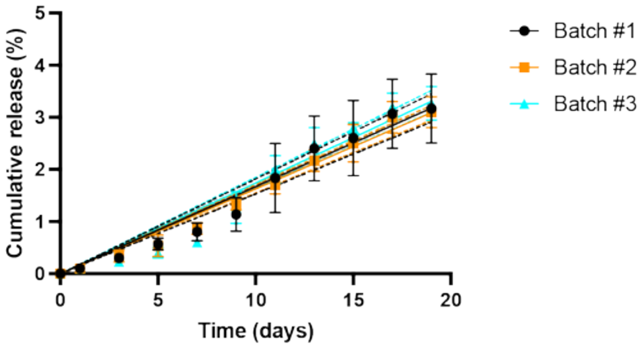

Equation for Zero-order with delayed drug release model: $M_t = k_0 t$

	Batch #1	Batch #2	Batch #3
k_0	0.1671	0.1625	0.1740
R squared	0.9869	0.9922	0.9705
Lower Level 95% Confidence Interval	0.1529	0.1550	0.1630
Upper Level 95% Confidence Interval	0.1814	0.1700	0.1850

Figure S4. Quantification of the batch-to-batch variation in the microfluidics method for rhCCL22 release kinetics in terms of the zero-order with delayed release kinetics constant, k_0. rhCCL22-loaded PLGA microparticles were prepared by the microfluidics method. The region between dashed lines color coded with the corresponding legend depicts the 95% confidence interval region for each batch.
